# PlantFormer: a precise plant disease segmentation network with interactive backbone and global-anisotropic context aggregation

**DOI:** 10.3389/fpls.2026.1853571

**Published:** 2026-06-26

**Authors:** Cuibiao Feng, Yulong Fan, Xi Chen, Hui Wang

**Affiliations:** 1 Yiwu Industrial & Commercial College, Yiwu, China; 2 Jinhua City Key Laboratory of Intelligent Welding Technology for Robotics, Jinhua, China; 3 Zhejiang Normal University, Jinhua, China

**Keywords:** context aggregation, deep learning, plant disease segmentation, semantic segmentation, swin transformer

## Abstract

Precise plant disease segmentation in real-world agricultural environments presents challenges that general-purpose models often fail to address, primarily due to the anisotropic spread of lesions, blurred biological boundaries, and severe background dominance. To overcome these bottlenecks, this paper proposes PlantFormer, an end-to-end network that effectively integrates and adapts advanced architectural components to address these domain-specific issues. Specifically, PlantFormer employs an InteractSwin Backbone with a Cross-Level Fusion (CLF) module to preserve early pathological details. To model highly directional disease propagation, a GlobalAnisotropic Context Aggregation (GACA) neck utilizing strip pooling is introduced. Furthermore, a Semantic-Guided Fusion (SGF) decoder acts as a feature “boundary purifier” to suppress field noise, while a decoupled boundary-aware loss function explicitly shifts the optimization focus from healthy leaf regions to subtle necrotic transition zones. Comprehensive experiments demonstrate the effectiveness of our approach: PlantFormer achieves 41.78% mIoU on the complex PlantSeg dataset (unstructured field conditions) and 93.54% mIoU on the structured NLB dataset (vein-aligned lesions). It outperforms generalist models such as DeepLabV3+ and Segformer in key metrics like mIoU and mAcc. Despite these promising results, limitations remain, particularly regarding performance in scenarios with high-density, early-stage disease outbreaks, which will be the focus of future work.

## 1 Introduction

With the growing demand for precision agriculture, the use of computer vision and deep learning techniques for automated, high-precision plant disease detection has become a key research direction (Kamilaris and Prenafeta-Bold’u, 2018). Precise disease segmentation not only provides a pixel-level basis for the quantitative analysis of disease severity, thereby guiding targeted pesticide application and reducing overuse, but also effectively lowers agricultural production costs. It holds significant importance for ensuring food security and promoting sustainable agricultural development (Singh et al., 2021).

Early research on plant disease segmentation primarily relied on traditional image processing techniques and manually designed features (Otsu, 1979; Dhanachandra et al., 2015; Singh and Misra, 2016; Adams and Bischof, 1994; Kass et al., 1988). Although computationally inexpensive, these methods typically require extensive manual feature engineering and exhibit weak robustness in complex, real-world field environments. In recent years, deep learning methods, represented by Convolutional Neural Networks (CNNs) [e.g., FCN (Long et al., 2015), U-Net (Ronneberger et al., 2015), and DeepLabV3+ (Chen et al., 2018)] and Transformer-based architectures [e.g., Segformer (Xie et al., 2021)], have driven significant progress in dense prediction tasks.

Recently, several studies have significantly advanced agricultural disease segmentation based on DeepLabV3 architectures (Ding et al., 2025; Li et al., 2025; Pal et al., 2024). While these targeted modifications demonstrate robust feature extraction capabilities in agricultural contexts, they still face challenges in delicately balancing high-resolution boundary preservation with computational overhead, a specific gap that our PlantFormer addresses.

However, existing state-of-the-art general-purpose segmentation models often yield suboptimal results when directly applied to agricultural scenarios. The fundamental reason is that these models implicitly treat lesions as common discrete objects (like cars or animals), ignoring the fact that a plant lesion is essentially a biological spreading process. Consequently, plant disease segmentation faces three unique biological and environmental challenges that differentiate it from standard computer vision tasks:

Anisotropic Spread of Lesions: Unlike conventional objects with relatively regular shapes, many agricultural diseases (such as Northern Leaf Blight) spread along leaf veins, exhibiting highly elongated and continuous structural morphologies. Traditional square convolution kernels or simple global attention mechanisms struggle to capture this distinct anisotropic biological prior.

Blurred Biological Boundaries: A lesion is not a rigid object with hard edges. From the necrotic center to the healthy leaf tissue, there is a gradual transition zone characterized by chlorosis or water-soaked halos. This biological gradient is susceptible to confusion with highlights, reflections, and shadows, leading to significant boundary degradation.

Field Noise and Background Dominance: In natural field environments, varying illumination, overlapping foliage, and complex soil backgrounds substantially interfere with visual perception. More crucially, this results in a pronounced “foreground-background imbalance,” where abundant healthy leaf pixels disproportionately outweigh the critical but smaller disease features during model optimization.

Concurrently, the field has witnessed the emergence of massive foundation models [e.g., SAMbased architectures (Kirillov et al., 2023)] and powerful frameworks like Mask2Former (Cheng et al., 2022). These models have shown unprecedented capabilities in generic segmentation, driving recent trends in few-shot and zero-shot learning paradigms. However, their immense parameter counts and high computational demands represent a fundamentally different paradigm, often hindering their direct deployment on resource-constrained agricultural edge devices. Rather than competing in the open-vocabulary massive model space, PlantFormer is positioned as distinct from, yet complementary to, these approaches: it serves as a fully-supervised, domain-specific, and computationally accessible mid-weight alternative (32.1M parameters) tailored for environments where precise biological boundary delineation is paramount.

To address these agricultural domain-specific bottlenecks, we propose PlantFormer. While integrating established components—such as Swin Transformer, FPN, strip pooling, and CARAFE++—our core technical novelty lies in the non-trivial structural adaptation and synergy of these modules to explicitly model plant pathology priors. Rather than a simple concatenation of existing mechanisms, PlantFormer is carefully wired to translate specific biological constraints into targeted architectural designs:

Global-Anisotropic Context Aggregation (GACA): To model the anisotropic spread of diseases, we introduce the GACA module. By explicitly incorporating strip pooling mechanisms, GACA is tailored to effectively align with the physical morphology of vein-aligned lesions (e.g., stripes). This approach aligns more closely with plant pathology priors than simply expanding the receptive field isotropically.

Semantic-Guided Fusion (SGF) as a Boundary Purifier: To tackle the blurred biological boundaries, we design the SGF module to act as a feature “boundary purifier.” It leverages high-level semantics to actively suppress pseudo-edge noise (e.g., soil and reflections) in shallow features, selectively preserving genuine lesion boundary details.

Decoupled Boundary-Aware Loss: Observing the pronounced background dominance in agricultural imagery, we propose a decoupled boundary-aware loss. This mechanism explicitly shifts the model’s optimization focus away from the predominantly “healthy regions” toward the subtle “necrotic edge transitions,” effectively mitigating the class imbalance.

Furthermore, an InteractSwin Backbone with a Cross-Level Fusion (CLF) mechanism is utilized to achieve early interaction between deep semantics and shallow details at the early stages of feature extraction.

To evaluate the robustness of our approach in authentic field environments, we conduct comprehensive experiments on two carefully selected public datasets: PlantSeg and NLB. These datasets represent two contrasting morphological profiles in nature. PlantSeg represents “unstructured and complex field conditions,” evaluating the model’s robustness against complex backgrounds and varying illumination. Conversely, NLB represents “structured, anisotropic morphologies,” demonstrating GACA’s capability in capturing slender, vein-aligned lesions.

The results demonstrate that PlantFormer achieves highly competitive performance across these diverse morphological profiles, showing clear improvements over generalist models like DeepLabV3+ and Segformer. This highlights its robustness against unstructured and anisotropic lesions in the wild, providing a reliable visual engine for future precision agricultural interventions.

## 2 Materials and methods

To systematically address the unique biological and environmental challenges in agricultural imagery—namely the anisotropic spread of lesions, blurred biological boundaries, and pronounced background dominance—we propose PlantFormer, a novel end-to-end network tailored for precise plant disease segmentation. Building upon a generic Encoder-Neck-Decoder architecture, PlantFormer goes beyond standard implementations by contextualizing established mathematical and structural operations (such as Swin-based interaction, strip pooling, and weighted boundary loss) within the agricultural domain. Each component is specifically adapted and synergized to explicitly address the aforementioned plant pathology priors. This section elaborates on the representative datasets chosen, the biology-driven architecture of the model, the design motivation of its core components, and the customized decoupled loss function.

### 2.1 Datasets and preprocessing

#### 2.1.1 Datasets description

To rigorously evaluate the model’s performance in authentic agricultural environments, we specifically focus on high-quality datasets captured in the wild. We explicitly excluded widely used laboratory-controlled datasets (such as PlantVillage) as their uniform backgrounds do not reflect the real-world complexities our network aims to solve. Furthermore, while datasets like PlantDoc contain field images, their segmentation masks are known to exhibit significant noise, rendering them unsuitable for precise boundary-level evaluation. Therefore, to demonstrate the robustness of PlantFormer across a wide spectrum of agricultural scenarios, our evaluation is anchored on two carefully selected datasets representing distinct morphological types:

PlantSeg Dataset (Wei et al., 2024): Representing “unstructured and complex field conditions”. This large-scale dataset contains over 11,400 images collected in real-world field environments, covering 115 diseases across 34 crop types. Its high complexity—driven by dynamic illumination, overlapping leaves, and complex soil backgrounds—provides a robust platform for testing the model’s resistance to field noise and severe foreground-background imbalance.

NLB (Northern Leaf Blight) Dataset (Prashanth et al., 2023): Representing “structured, anisotropic morphologies”. This public dataset focuses on Northern Leaf Blight in maize under field conditions (1,000 images). Its lesions spread strictly along leaf veins, exhibiting distinctly slender and continuous structural forms. This dataset serves as a suitable testbed to evaluate the model’s capability in capturing anisotropic biological priors.

For dataset partitioning, we adopted specific strategies based on the characteristics of each dataset. For the PlantSeg dataset, we strictly followed the official benchmark setting, which utilizes an 80/20 train/test split. To perform necessary hyperparameter tuning while preventing data leakage, we further deterministically partitioned the official 80% training set into a sub-training and validation set using a fixed random seed, ensuring the official 20% test set remained untouched for the final evaluation. For the NLB dataset, which lacks an official benchmark split, we maintained a consistent random partitioning protocol across all evaluated baseline models to ensure a strictly fair, applesto-apples comparison.

#### 2.1.2 Data preprocessing and augmentation

To mitigate overfitting and improve the robustness of the model against field variations, standard data augmentation strategies were applied:

- Geometric Transformations: Random horizontal/vertical flips, and random rotations (within ±30°) to simulate varied camera angles.
- Photometric Distortions: Random adjustments to brightness, contrast, and saturation to simulate variable lighting conditions in open fields.
- Normalization: Input images were resized to 512 × 512 pixels and normalized using ImageNet statistics to accelerate convergence.

### 2.2 Overall architecture

**Figure 1:**
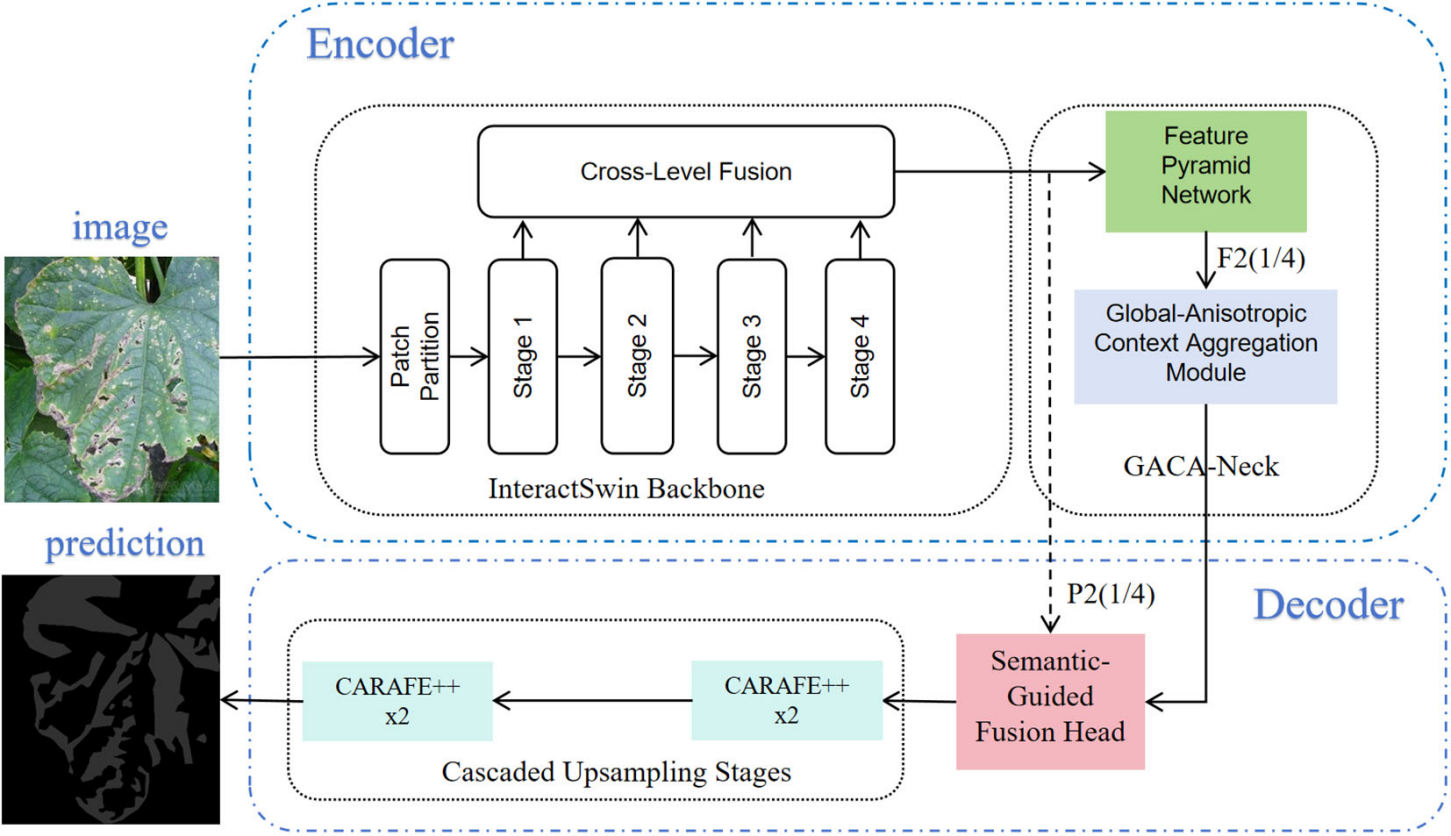
Overall architecture of PlantFormer. It illustrates the complete data flow mapping plant pathology priors to network designs, including the InteractSwin Backbone, the GACA neck for anisotropic spread, and the SGF Decoder for purifying blurred biological boundaries.

The overall architecture of PlantFormer, depicted in **Figure 1**, consists of three core components explicitly mapped to the aforementioned agricultural challenges:

Encoder: An InteractSwin Backbone equipped with a Cross-Level Fusion (CLF) module, designed to facilitate early interaction between deep semantics and spatial details, reducing the loss of subtle pathological features.

Neck: A combination of a standard FPN (Lin et al., 2017a) and our novel Global-Anisotropic Context Aggregation (GACA) module. GACA acts as the core engine to model the highly directional propagation of plant diseases.

Decoder: Led by the Semantic-Guided Fusion (SGF) Head and cascaded with a CARAFE++ (Wang et al., 2021) upsampling operator, functioning as a “boundary purifier” to reconstruct accurate lesion contours.

First, the input image passes through the InteractSwin backbone, generating interactively enhanced feature pyramids *P*_2_*,P*_3_*,P*_4_*,P*_5_. The FPN then performs top-down fusion, producing features *F*_2_*,F*_3_*,F*_4_*,F*_5_. Crucially, *F*_2_ is fed into the GACA module for deep anisotropic context enhancement, yielding *F*_GACA_. Finally, the SGF module in the decoder receives the semanticrich *F*_GACA_ and the detailed *P*_2_. After selectively filtering out background noise, two cascaded CARAFE++ modules restore the feature map to the original resolution, generating the precise disease prediction map.

### 2.3 Encoder: interactive backbone and cross-level fusion

In typical backbones, the linear bottom-up extraction naturally creates a “semantic gap”: deep features lack spatial resolution, while shallow features are often affected by background noise. To preserve subtle pathological details from the early stages of the network, the Cross-Level Fusion (CLF) module is embedded between adjacent Swin Transformer (Liu et al., 2021) stages.

**Figure 2:**
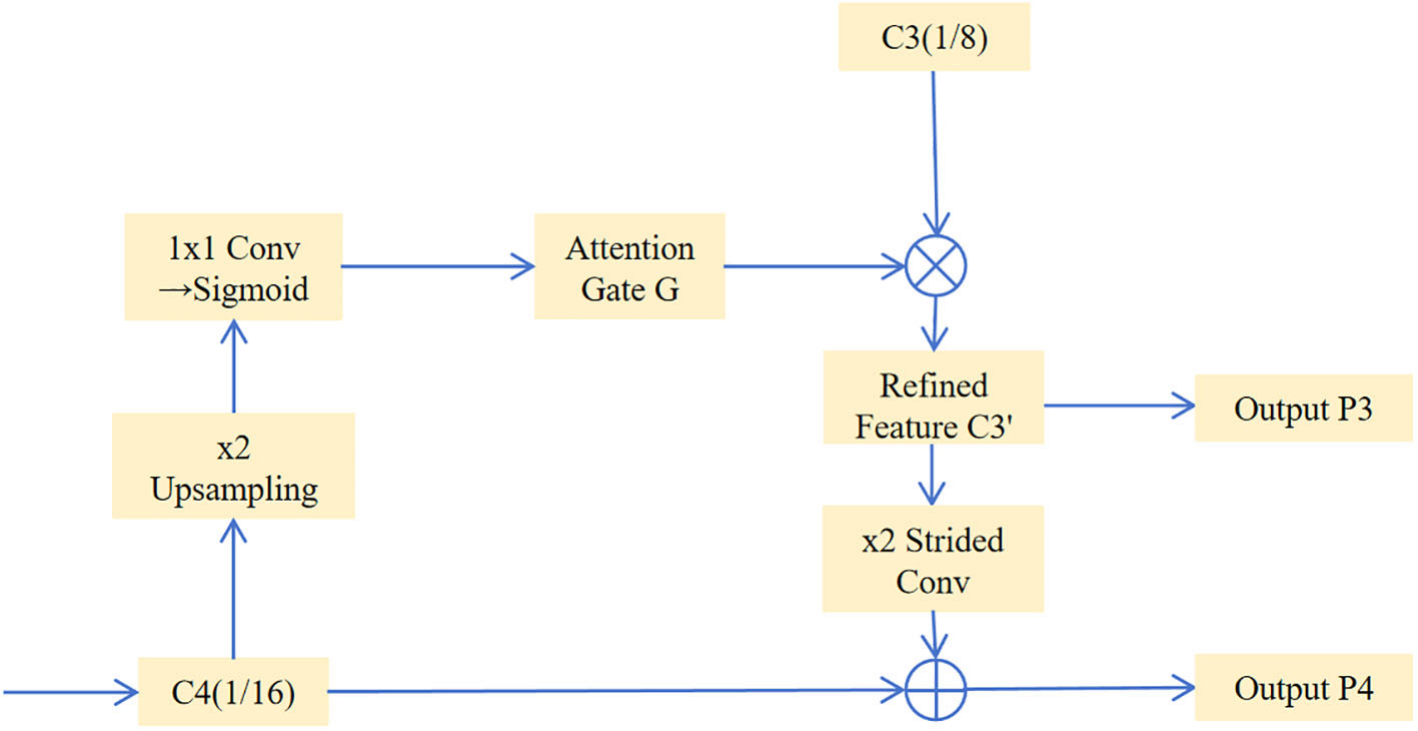
Schematic diagram of the Cross-Level Fusion (CLF) module unit, utilizing top-down semantic guidance to preserve pathological details alongside bottom-up supplementation.

Operating in a top-down, cascaded manner, CLF ensures that the information flow is continuously refined. As illustrated in **Figure 2** (using *C*_4_ and *C*_3_ as an example), the deeper semantic feature generates a spatial attention gating signal to pinpoint key pathological regions, highlighting them in the shallow feature *C*_3_. Simultaneously, the refined shallow details are injected back into the deep feature path. This continuous interaction ensures that the output pyramids (*P*_2_ to *P*_5_) possess a robust foundation for subsequent anisotropic mining.

### 2.4 Neck: FPN and GACA module

**Figure 3:**
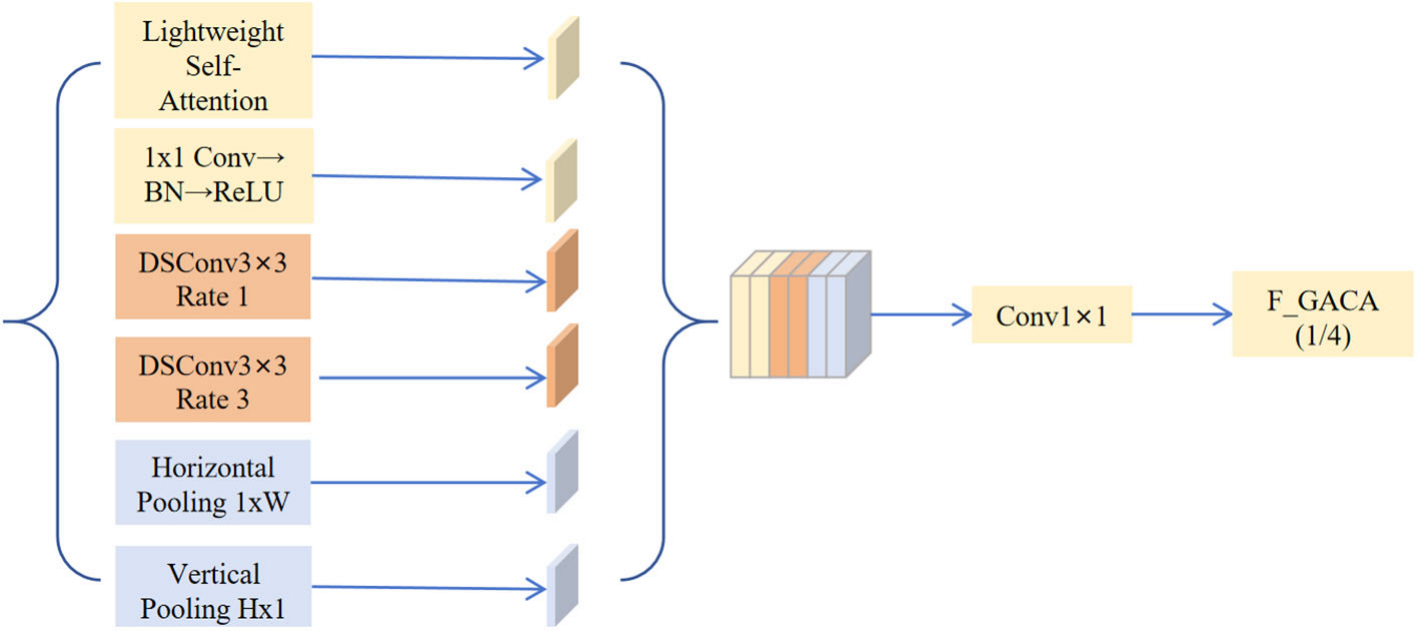
Structure of the GACA module. The strip pooling branch is explicitly designed to capture the anisotropic spread of vein-aligned lesions.

To specifically address the biological characteristic of anisotropic spread—where diseases like Northern Leaf Blight propagate structurally along leaf veins, forming slender stripes—we introduce the Global-Anisotropic Context Aggregation (GACA) module (**Figure 3**). The inclusion of specific operations within GACA is a tailored solution to better capture the physical morphology of these vein-aligned lesions.

GACA systematically captures context through four complementary dimensions:

Anisotropic Context (The Core Design): To target slender, stripe-like diseases, we adapt the Strip Pooling mechanism (Hou et al., 2020). By deploying horizontal (1xW) and vertical (Hx1) long stripshaped pooling kernels, we repurpose this mechanism to aggregate directionally strong contextual features. This aligns with plant pathology priors, compensating for the limitations of standard square convolution kernels, which often struggle to encapsulate the continuous spread of vein-aligned pathological structures.

Multi-scale Local Context: Parallel depth-wise separable convolutions (DSConv3x3, rate=1, 3) adapt to locally varying sizes of early-stage disease spots.

Global Dependencies: A Lightweight Self-Attention module establishes long-range relationships between distant infected regions across the leaf surface.

Pixel-level Detail Preservation: A standard 1x1 Conv branch prevents the microscopic textures of necrotic spots from being smoothed out.

By fusing these four heterogeneous branches, GACA produces a specialized feature map (*F*_GACA_) that mathematically represents the biological spread of the disease.

### 2.5 Decoder: semantic-guided fusion as a boundary purifier

Traditional decoders often employ direct fusion methods (e.g., simple concatenation or addition), which can be problematic when dealing with blurred biological boundaries. Because a lesion’s transition zone (chlorosis or water-soaked halos) lacks hard edges, directly fusing shallow features inadvertently introduces background noise (soil, shadows, and leaf reflections) into the decoding path, compromising the boundaries.

**Figure 4:**
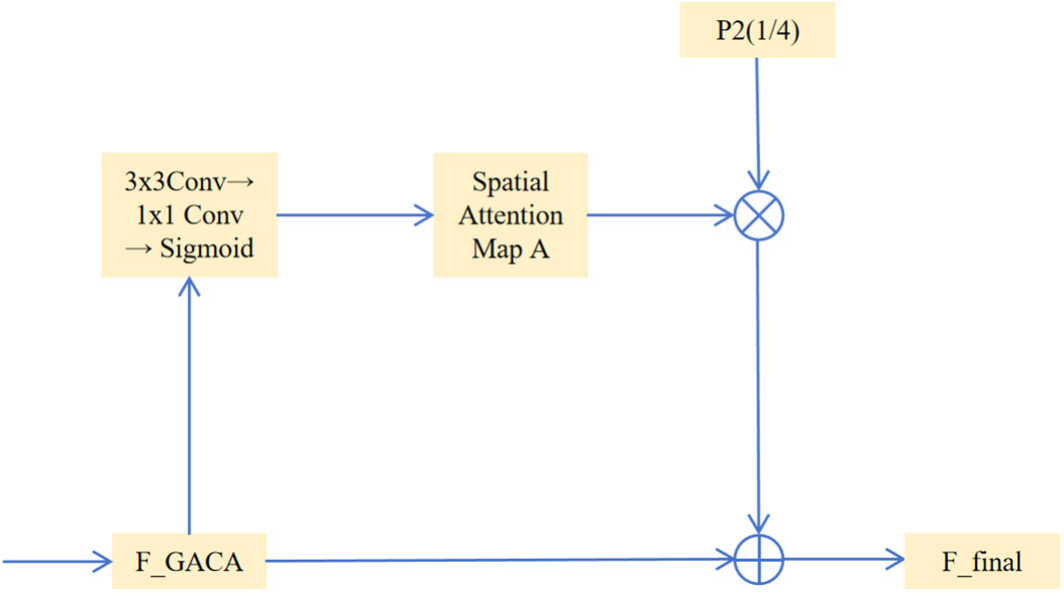
Internal structure of the SGF module, functioning as a boundary purifier. Deep semantics are used to suppress field noise (e.g., soil, reflections) in shallow features.

To tackle this, the Semantic-Guided Fusion (SGF) Head (**Figure 4**) is designed based on attentionbased fusion concepts to act as a “boundary purifier.” SGF leverages the global semantic perspective of the deep feature (*F*_GACA_) to generate a Spatial Attention Map. This map acts as a spatial filter, suppressing pseudo-edge noise in the shallow detail feature (*P*_2_). Consequently, only the refined lesion boundary details are permitted to pass through and fuse with the semantics.

Following purification, cascaded CARAFE++ (Wang et al., 2021) modules dynamically predict content-aware reorganization kernels to perform progressive upsampling. Interleaved with 3x3 convolutions to mitigate checkerboard artifacts, this pipeline supports the precise recovery of the biological gradient zones, yielding more refined segmentation contours.

### 2.6 Loss function and training strategy

#### 2.6.1 Decoupled, boundary-aware hybrid loss function

Agricultural imagery inherently suffers from extreme long-tail distributions and background dominance. A standard loss function is often dominated by the preponderance of healthy leaf pixels, causing the subtle necrotic boundary transitions to be neglected during optimization.

To redirect the model’s optimization focus from “predominantly healthy regions” to “subtle lesion edges,” we propose a decoupled, boundary-aware hybrid loss function (*L*_total_). The decoupled, boundary-aware hybrid loss is formulated in Equations 1–4, and the evaluation metrics are defined in Equations 5–8. This hybrid loss adapts and combines standard loss formulations to decompose the optimization into two sub-goals:

(1) $$L_{\text{total}}=L_{\text{region}}+\lambda L_{\text{boundary}}$$

where *λ* balances the two sub-tasks.

The Region Loss (Lregion) tackles the class imbalance by combining Dice Loss (Milletari et al., 2016) and Focal Loss (Lin et al., 2017b).

(2) $$L_{\text{region}}=L_{\text{Dice}}+L_{\text{Focal}}$$

This combination directly optimizes the intersection over union while dynamically down-weighting the easily classified healthy leaf pixels.

The Boundary Loss (*L*_boundary_) is specifically targeted at overcoming the blurred biological boundaries. A Boundary-aware Weighted BCE Loss is adopted. Prior to training, a boundary weight map is generated using a distance transform for each mask:

(3) $$w_{i}=1+\alpha e^{-\beta d_{i}}$$

where *d_i_*is the distance from pixel *i* to the nearest true lesion boundary. This formulation assigns exponentially higher weights to pixels residing in the subtle transition zones. In our implementation, the coefficients for the boundary weight map were empirically set to *α* = 10.0 and *β* = 0.1. The loss is computed as:

(4) $$L_{\text{boundary}}=-\frac{1}{N}\sum_{i=1}^{N}w_{i}\left[y_{i}\log\;(p_{i}\right)+\left(1-y_{i}\right)\log\;(1-p_{i})]$$

This mechanism guides the gradients to proportionately penalize errors along the fading lesion edges. It forms an effective “architecture-loss synergy” with the SGF boundary purifier, jointly driving accurate boundary delineation.

#### 2.6.2 Progressive training strategy

Given the background dominance, optimizing *L*_total_ from scratch can be challenging, as background gradients tend to dominate early epochs. Thus, a Progressive Two-Stage Training Strategy is employed:

Stage 1: Region-Aware Pre-training. The boundary loss is frozen (*λ* = 0). The model uses only *L*_region_ to overcome the initial background suppression, learning the macro-location of the disease.

Stage 2: Boundary Refinement. Building on Stage 1, the model is fine-tuned with the complete decoupled loss (*λ >* 0) at a lower learning rate. With the macro-regions established, the boundary loss effectively refines the biological gradient zones.

## 3 Results

In this section, the performance of the proposed PlantFormer is systematically evaluated. Beyond reporting numerical improvements, the following experiments and ablation studies analyze how each architectural design addresses the identified biological and environmental bottlenecks (anisotropic spread, blurred biological boundaries, and background dominance). Finally, quantitative and qualitative comparisons with recent competitive general-purpose segmentation models are conducted on the PlantSeg and NLB datasets to demonstrate PlantFormer’s robustness across different morphological types.

### 3.1 Experimental setup

As outlined in Section 2, experiments were conducted on PlantSeg (representing unstructured field conditions) and NLB (representing structured, anisotropic morphologies). Testing the model on these two contrasting profiles serves as an indicator of its applicability to a variety of crop diseases.

#### 3.1.1 Evaluation metrics

Two standard dense prediction metrics, Mean Intersection over Union (mIoU) and Mean Pixel Accuracy (mAcc), were initially utilized to evaluate model performance:

(5) $$mIoU=\frac{1}{N_{c}}\overset{N_{c}}{\underset{c=1}{\sum }}\frac{TP_{c}}{TP_{c}+FP_{c}+FN_{c}}$$

(6) $$mAcc=\frac{1}{N_{c}}\overset{N_{c}}{\underset{c=1}{\sum }}\frac{TP_{c}}{TP_{c}+FN_{c}}$$

where *N_c_*is the total number of classes, and *TP_c_*, *FP_c_*, *FN_c_*represent the true positive, false positive, and false negative pixels for class *c*, respectively. Note that in our formulation, mAcc calculates the proportion of correctly identified positive pixels out of all actual positive pixels for each class, which is mathematically equivalent to the Macro-Recall (Sensitivity) metric.

Furthermore, to rigorously validate the model’s capability in preserving fine-grained lesion structures and delineating blurred biological boundaries, we introduced additional region-level and boundary-specific metrics: Dice Score, Precision, Boundary IoU, and Boundary F1 Score. The formulas for Dice and Precision are as follows:

(7) $$Dice=\frac{1}{N_{c}}\overset{N_{c}}{\underset{c=1}{\sum }}\frac{2\times TP_{c}}{2\times TP_{c}+FP_{c}+FN_{c}}$$

(8) $$Precision=\frac{1}{N_{c}}\overset{N_{c}}{\underset{c=1}{\sum }}\frac{TP_{c}}{TP_{c}+FP_{c}}$$

For the boundary-specific metrics (Boundary IoU and Boundary F1), we strictly followed standard contour-based evaluation protocols. Specifically, these metrics are computed by applying a morphological dilation with a defined distance threshold (pixel tolerance) to extract the contour regions of both the ground truth and the predicted masks. By calculating the Intersection over Union and F1 score strictly within these dilated contour regions, this approach explicitly evaluates errors along the fading lesion edges, providing a more accurate reflection of the model’s capability in boundary delineation compared to global region metrics.

#### 3.1.2 Implementation and reproducibility details

All experiments were implemented using the PyTorch framework. To ensure the reliability of dataset partitioning, a fixed random seed was utilized. Furthermore, to ensure statistical robustness, all core ablation studies—including both the architectural components and the loss function variants—were repeated across three different random seeds, with results reported as mean ± standard deviation.

The models were trained on a hardware platform equipped with dual NVIDIA RTX 4090 GPUs. During training, all backbone networks (except U-Net) were initialized with ImageNet-1K pretrained weights, and input images were strictly resized to 512 × 512 pixels. For the optimization process, we utilized the AdamW optimizer with a batch size of 4 per GPU, a weight decay of 0.01, and an initial learning rate of 6 × 10−^5^, which was subsequently decayed using a polynomial schedule. The optimization process ran for a total of 320,000 iterations. Following the progressive two-stage training strategy, Stage 1 (Region-Aware Pre-training) was conducted for the initial 200,000 iterations. Subsequently, Stage 2 (Boundary Refinement) was executed for the remaining 120,000 iterations, with the loss function balance parameter (*λ*) activated and set to 1.0. Under these explicit configurations, the total training time was approximately 24 hours. These detailed hyperparameter settings ensure that the experimental conditions are transparent and reproducible.

### 3.2 Ablation studies

To validate the necessity of injecting plant pathology priors into the network architecture, we progressively added the proposed modules to a baseline model (Swin-T + FPN + Standard Decoder). The results are detailed in **Table 1**.

**Table 1:** Ablation study of the core biology-driven architectural components.

Model configuration	mIoU (%)	Gain
Baseline (Swin-T + FPN + Standard Decoder)	36.41 ± 0.26	–
Baseline + CLF (Interactive Backbone)	38.78 ± 0.18	+2.37
Baseline + CLF + GACA (Anisotropic Engine)	39.95 ± 0.23	+1.17
**PlantFormer** (Baseline + CLF + GACA + SGF)	**40.94 ± 0.17**	**+0.99**

Results are reported as mean ± standard deviation across three independent runs. The overall improvement of PlantFormer over the baseline is statistically significant (*p<* 0.05).

Bold values indicate the best performance among the compared configurations.

To validate the statistical significance of these architectural improvements, a paired t-test was conducted between the baseline and the proposed PlantFormer across the repeated runs. The test yielded a p-value of less than 0.05, confirming that the overall performance gain from our biology-driven designs is statistically significant.

The quantitative gains map directly to the resolution of specific agricultural bottlenecks:

- Securing Early Details (CLF): The introduction of the CLF module yielded a 2.37% mIoU increase. This demonstrates that early top-down semantic intervention successfully prevents the loss of minuscule pathological features at the source, overcoming the “semantic gap” of linear backbones.
- Capturing Anisotropic Spread (GACA): Integrating the GACA module further boosted the mIoU by 1.17%. This quantitative gain supports our core biological hypothesis: explicitly modeling the highly directional spread of lesions (using strip pooling) is more effective than simply expanding the receptive field with standard isotropic convolutions. Specifically, compared to isotropic context aggregators like ASPP, the selected strip pooling mechanism is hypothesized to align more closely with the highly directional, vein-aligned morphology of agricultural lesions, although a direct empirical comparison with ASPP-style alternatives remains a direction for future work (see Section 4).
- Purifying Blurred Boundaries (SGF): Replacing the standard decoder with the SGF “boundary purifier” provided an additional 0.99% gain, pushing the mIoU to 40.94%. This indicates that leveraging high-level semantics to actively suppress soil and reflection noise in shallow features is crucial for restoring biological transition zones. Compared to simple addition or concatenationbased fusion strategies, SGF is designed to more effectively suppress pseudo-edge noise by leveraging semantic guidance as a spatial filter, though a direct empirical comparison with these fusion alternatives is left for future investigation (see Section 4). Furthermore, the cascaded CARAFE++operator was selected over standard bilinear interpolation or transposed convolution to explicitly prevent the degradation of fine details and checkerboard artifacts during upsampling.

#### 3.2.1 Effectiveness validation of custom loss function

Furthermore, we evaluated the impact of explicitly shifting the optimization focus to mitigate background dominance. To validate the effectiveness of our proposed loss function, **Table 2** compares our decoupled boundary-aware loss (*L*_boundary_) against a strong standard baseline (BCE+Dice loss) and the Dice+Focal combination.

**Table 2:** Ablation study of the decoupled boundary-aware hybrid loss function.

Model configuration	mIoU (%)	Boundary F1 (%)
PlantFormer + Standard BCE+Dice Loss	40.18 ± 0.15	47.39 ± 0.25
PlantFormer + Dice+Focal Loss	40.94 ± 0.17	48.56 ± 0.24
PlantFormer + Dice+Focal+ *L_boundary_* (Final)	**41.78 ± 0.14**	**49.82 ± 0.21**
Gain (Final vs. Dice+Focal)	+0.84	+1.26

Results are reported as mean ± standard deviation across three independent runs.

Bold values indicate the best performance among the compared configurations.

The integration of *L*_boundary_ secured a final average improvement of 0.84% in mIoU over the Dice+Focal setup, alongside a more pronounced 1.26% increase in the Boundary F1 score. Similar to the architectural ablations, a paired t-test confirmed that these gains from the decoupled boundaryaware loss are statistically significant (*p<* 0.05). Furthermore, while boundary-distance metrics like Hausdorff loss exist, they can be unstable on highly fragmented plant lesions; our decoupled loss provides stable and explicit gradient guidance to polish the fading edges, yielding geometrically more accurate contours.

### 3.3 Comparison with recent competitive methods

We compared PlantFormer with several mainstream and competitive general-purpose semantic segmentation models (including CNN-based DeepLab series and Transformer-based Segformer).

#### 3.3.1 Results on the PlantSeg dataset (in-the-wild chaos)

As shown in **Table 3**, PlantFormer achieved the highest region-level accuracy with 41.78% mIoU and 60.05% Dice. More importantly, when evaluating fine-grained morphological delineation, PlantFormer vastly outperformed established baselines, achieving a Boundary F1 of 49.82% and Boundary IoU of 33.68%. The significant gap between PlantFormer and standard generalist models (like DeepLabV3+ with a Boundary F1 of only 31.20%) highlights the severe limitations of treating field agricultural imagery as standard CV tasks. General models often experience severe boundary degradation under chaotic illumination and complex background interference, whereas PlantFormer’s noise-suppression mechanisms (SGF) maintain robust contour fidelity.

**Table 3:** Performance comparison on the unstructured PlantSeg dataset.

Method	Backbone	mIoU (%)	mAcc/Recall (%)	Dice (%)	Precision (%)	Bound. IoU (%)	Bound. F1 (%)
U-Net	U-Net (s5-d16)	3.14	3.82	6.12	5.85	0.00	0.00
FCN	ResNet-101	9.63	13.28	17.65	18.20	1.35	2.72
DeepLabV3+	ResNet-101	27.18	42.29	43.51	41.83	18.73	31.20
Segformer	MiT-B2	40.66	55.24	58.74	59.38	31.14	47.10
PlantFormer (Ours)	InteractSwin-T	41.78	56.13	60.05	60.82	33.68	49.82

Regarding the evaluation of Boundary IoU and Boundary F1,westrictly adhere to a handling rule that assigns a zero score to any individual degenerate mask where no valid contour is detected.This image-level penalization explains the exceptionally low or near-zero contour metrics observed for early baselines on highly complex datasets.

It is important to note that very recent advanced architectures, such as SegNeXt-MSCANL (Guo et al., 2022), have reported higher overall mIoU (e.g., 44.52%) on the public PlantSeg benchmark. However, this absolute accuracy comes with a substantial computational cost. SegNeXtLarge requires approximately 50.1M parameters and 69.8 GFLOPs. In contrast, PlantFormer acts as a highly cost-effective alternative, achieving 41.78% mIoU while requiring significantly fewer computational resources (32.1M parameters, 38.7 GFLOPs). This favorable efficiency-accuracy trade-off makes PlantFormer highly suitable for practical agricultural deployment where hardware constraints are a primary consideration.

#### 3.3.2 Results on the NLB dataset (highly structured morphology)

**Table 4** presents the results on the highly structured NLB dataset. While general models perform better here due to cleaner backgrounds, PlantFormer demonstrates competitive segmentation performance and improved boundary delineation on the evaluated datasets, reaching 93.54% mIoU and a remarkable 90.20% Boundary F1, while offering a favorable efficiency-accuracy trade-off. This confirms that even when the background is relatively clean, lacking the architectural capacity to perceive anisotropic spread prevents general models from accurately encapsulating the boundaries of slender, vein-aligned pathologies.

**Table 4:** Performance comparison on the highly structured NLB dataset.

Method	Backbone	mIoU (%)	mAcc/Recall (%)	Dice (%)	Precision (%)	Bound. IoU (%)	Bound. F1 (%)
U-Net	U-Net (s5-d16)	77.43	83.63	87.35	86.82	58.45	73.88
FCN	ResNet-101	88.51	92.63	93.82	94.15	79.20	88.45
Segformer	MiT-B2	89.29	93.85	94.48	94.62	76.35	86.50
DeepLabV3+	ResNet-101	90.35	94.34	95.12	95.27	78.58	87.90
PlantFormer (Ours)	InteractSwin-T	93.54	97.08	96.85	96.11	82.37	90.20

### 3.4 Qualitative results analysis

**Figure 5:**
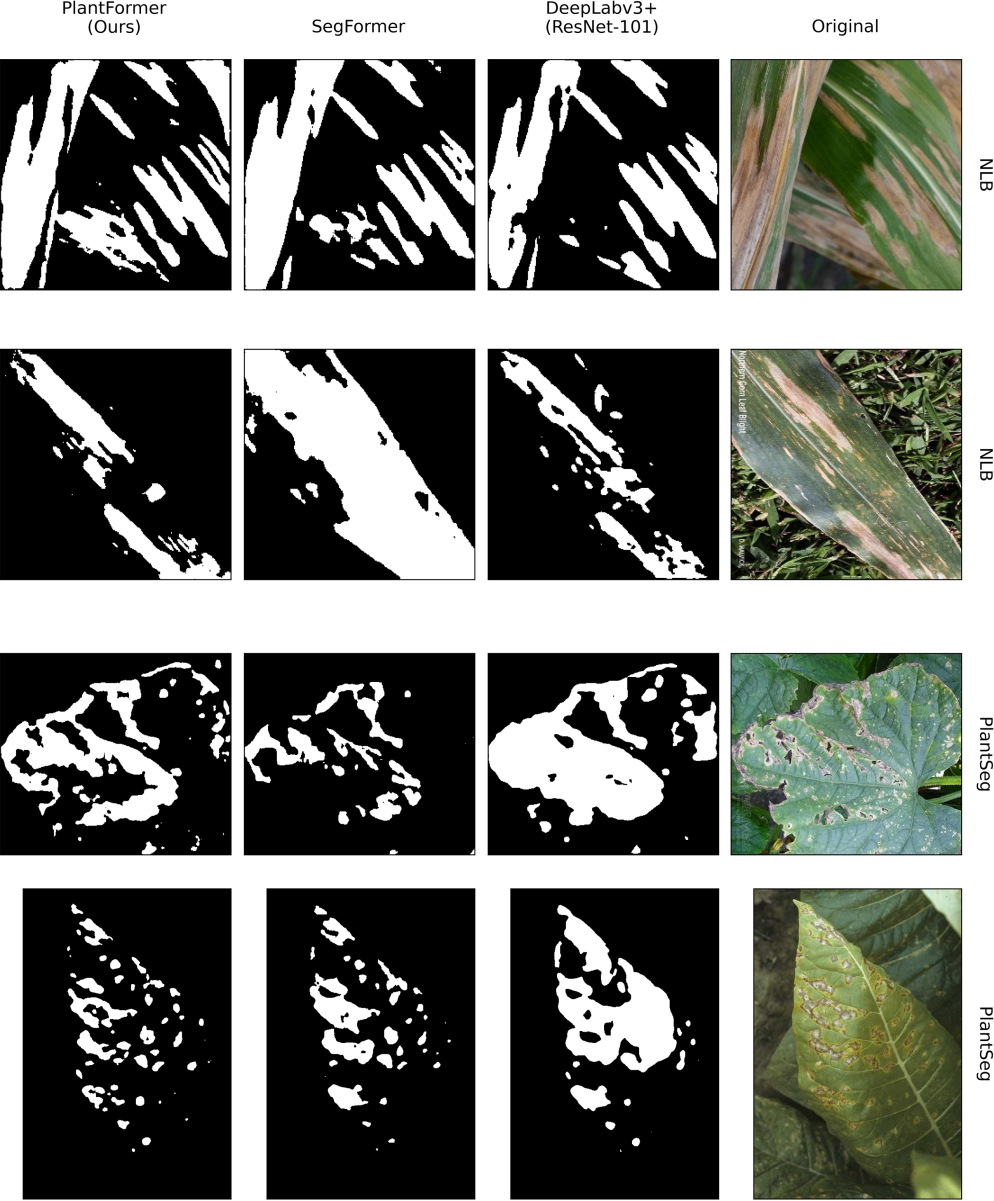
Qualitative comparison. From top to bottom: Input, DeepLabV3+ (ResNet-101), Segformer, and PlantFormer (Ours). Notice PlantFormer’s superiority in maintaining continuous anisotropic structures and suppressing background noise.

To intuitively illustrate why PlantFormer outperforms generalist models, we visualize the segmentation masks of challenging samples (**Figure 5**).

Overcoming Anisotropic Structural Breaks (NLB): The primary challenge in NLB is the long, continuous, spindle-shaped lesions. CNN-based models (DeepLab series) produce fragmented and disconnected masks because standard square convolutions lack the directional receptive field required to track vein-aligned spreading. Conversely, PlantFormer (empowered by GACA’s strip pooling) generates highly continuous masks that preserve the structural integrity of the entire pathological process.

Resisting Background Dominance and Noise (PlantSeg): In the PlantSeg samples characterized by biological blurred boundaries and severe background noise, DeepLabV3+ and Segformer suffer from over-segmentation (classifying shadows or soil as lesions). PlantFormer’s predictions are substantially cleaner. This visually corroborates that the SGF module successfully filtered out pseudo-edge noise before decoding.

### 3.5 Visualization and interpretability analysis

**Figure 6:**
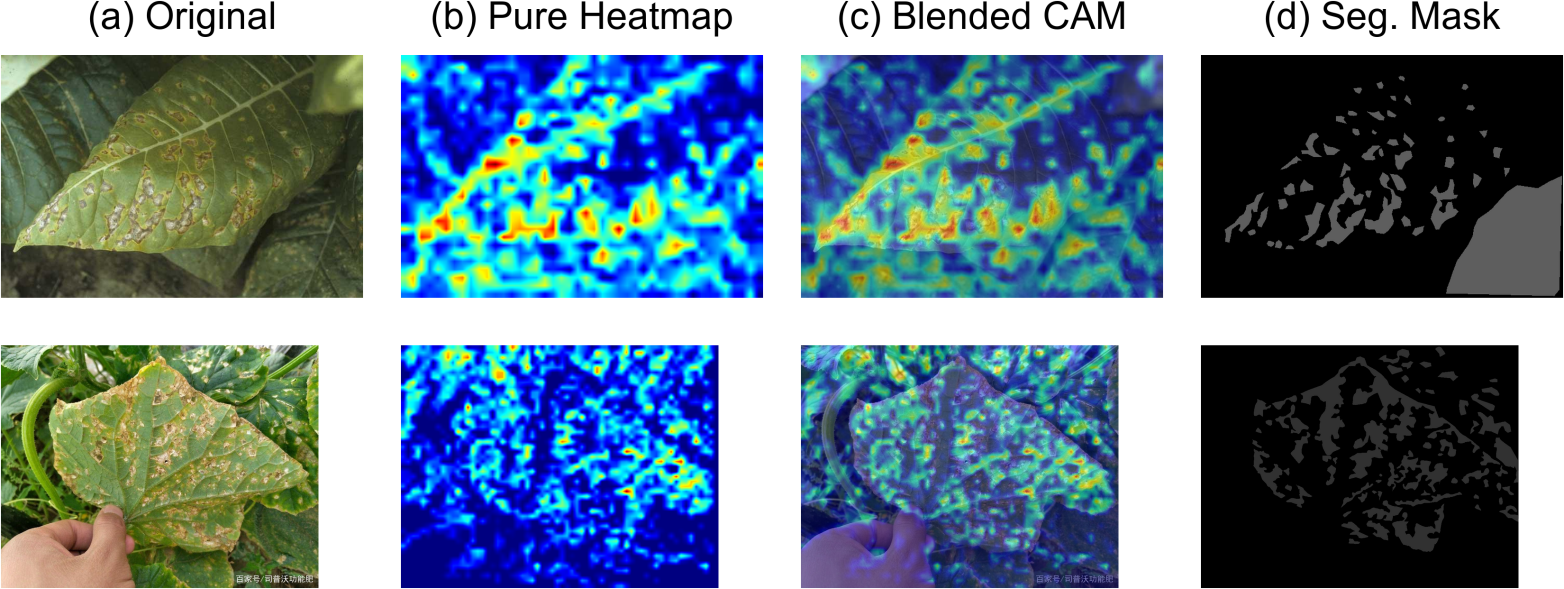
Grad-CAM feature activation visualizations. **(a)** Original leaf images; **(b)** pure heatmaps; **(c)** blended CAM visualizations; **(d)** predicted segmentation masks. The heatmaps and blended CAMs reveal that PlantFormer's attention highly conforms to the morphological spread of the disease, largely ignoring surrounding healthy tissue and soil noise.

To dissect the internal logic of PlantFormer, we utilized Grad-CAM to visualize feature activations (**Figure 6**). The Pure Heatmap (b) and Blended CAM (c) reveal that PlantFormer’s attention highly conforms to the morphological spread of the disease, largely ignoring surrounding healthy tissue and soil noise. This interpretability confirms that the network has learned genuine plant pathology features rather than overfitting to background biases.

**Figure 7:**
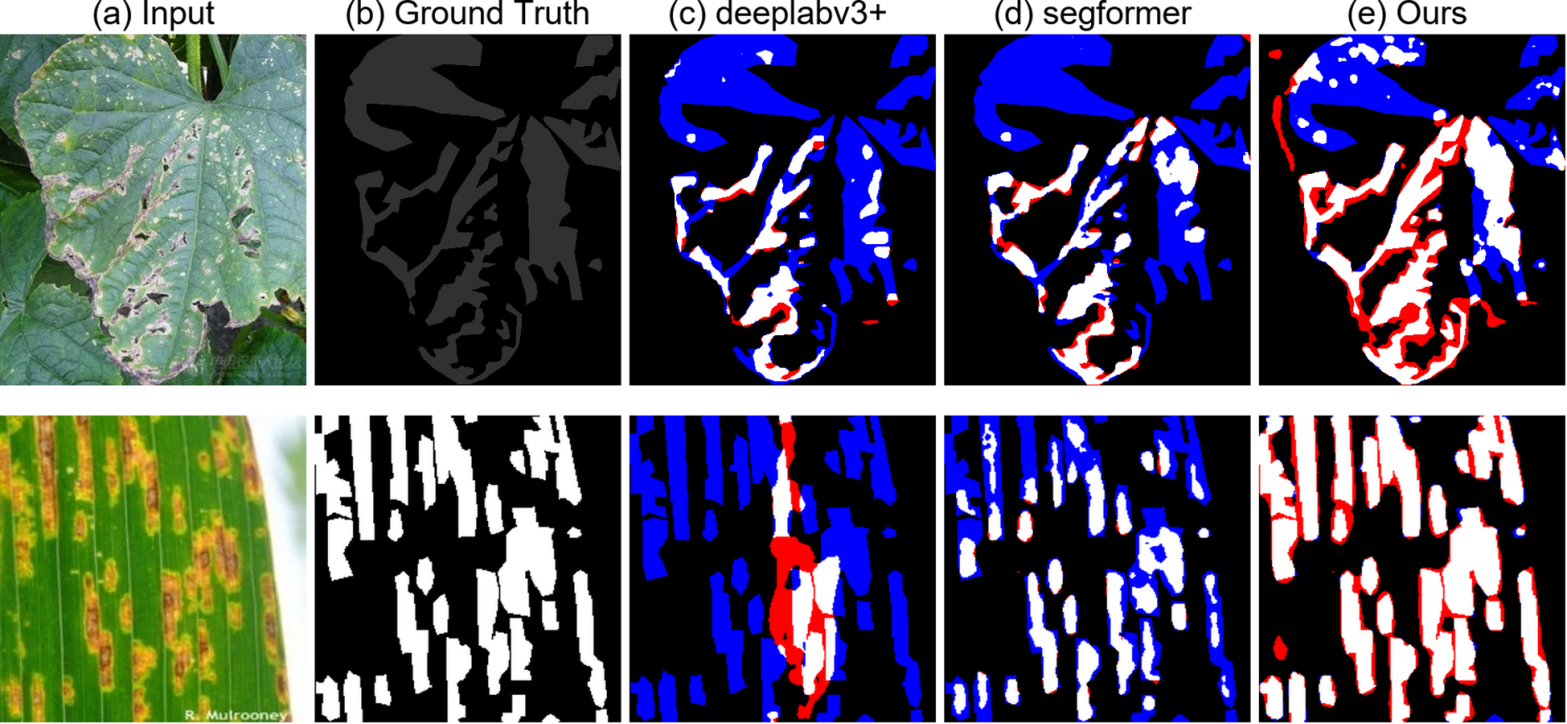
Close-up boundary comparison. **(a)** Original images; **(b)** ground-truth masks; **(c)** DeepLabV3+ results; **(d)** Segformer results; **(e)** PlantFormer results. General models like DeepLabV3+ and Segformer generate numerous false positives (red pixels) and false negatives (blue pixels) along the lesion borders due to direct feature fusion. PlantFormer exhibits a close alignment with the ground truth, providing visual proof of the architecture-loss synergy in handling biological gradient zones.

Furthermore, a close-up boundary comparison (**Figure 7**) reveals the limitations of general models in boundary delineation. Both DeepLabV3+ (c) and Segformer (d) generate numerous false positives (red pixels) and false negatives (blue pixels) along the lesion borders due to direct feature fusion. PlantFormer (e) exhibits a close alignment with the Ground Truth, providing visual proof of the architecture-loss synergy in handling biological gradient zones.

### 3.6 Model efficiency analysis

An effective agricultural AI model should balance precision with computational efficiency. We compared PlantFormer’s computational efficiency against key SOTA models (**Table 5**) using a single RTX 4090 GPU (512 × 512 inputs).

**Table 5:** Efficiency comparison.

Method	Backbone	mIoU (%)	Parameters (M)	FLOPs (G)	FPS
DeepLabV3+	ResNet-101	27.18	58.6	83.4	47.3
Segformer	MiT-B2	40.66	27.5	62.4	56.0
SegNeXt-MSCAN-L	MSCAN-L	44.52	~50.1	~69.8	–
PlantFormer (Ours)	InteractSwin-T	41.78	32.1	38.7	49.2

The mIoU, parameter count, and FLOPs for SegNeXt-MSCAN-L are reported from the published benchmark and were computed at 512 × 512 resolution. FPS is not available under our hardware setup. PlantFormer maintains efficient computation while achieving robust and high accuracy.

While delivering the highest segmentation accuracy, PlantFormer requires only 38.7 GFLOPs— less than half the computational load of DeepLabV3+, and significantly lighter than Segformer (62.4 GFLOPs). Tested on an NVIDIA RTX 4090, our model achieves competitive inference speeds (nearly 50 FPS), serving as a robust baseline compared to DeepLabV3+ and Segformer. This optimized computational core makes it suitable for high-throughput workstation-based agricultural diagnostic systems, and it shows strong potential for future adaptation and optimization for agricultural edge applications.

## 4 Discussion

### 4.1 Interpretability: biological priors over black-box overfitting

Deep learning models applied to agriculture are sometimes criticized for overfitting to datasetspecific biases rather than learning genuine pathological features. To assess whether PlantFormer’s performance stems from learning biological priors, we analyzed its internal feature activations (visualized in **Figure 6**).

The results reveal a “pathology-aware” attention pattern. Specifically, the GACA module encodes the anisotropic spread of diseases like Northern Leaf Blight, aligning the model’s focal regions along the leaf veins rather than irrelevant background textures. Furthermore, the SGF head functions effectively as a “boundary purifier.” As seen in **Figure 7**, while standard models may confuse soil reflections with necrotic halos, PlantFormer reduces false positive and false negative predictions along the transition zones. Crucially, this visual interpretability is now backed by robust quantitative validation. In plant pathology, accurate lesion area estimation and morphological integrity are key diagnostic criteria. Rather than relying solely on global metrics (mIoU), the significant improvements demonstrated by our newly introduced boundary-specific metrics (Boundary IoU and Boundary F1) and region-overlap metrics (Dice score) provide strict quantitative evidence. These metrics validate that PlantFormer rigorously preserves the precise area and structural contours of the lesions, mathematically confirming that the extracted features reflect biologically meaningful patterns.

### 4.2 Efficiency-accuracy trade-off

In precision agriculture, computational resources on edge devices (e.g., agricultural drones or Unmanned Ground Vehicles) are often constrained. A key aspect of PlantFormer is achieving a balance between capturing complex biological morphologies and maintaining computational efficiency.

As detailed in **Table 5**, PlantFormer achieves a higher mIoU compared to Segformer, with a moderate FLOPs requirement (38.7 G). Compared to heavier models like DeepLabV3+ (ResNet101), PlantFormer reduces the computational cost substantially while improving boundary accuracy. This balance is achieved through targeted strip pooling (GACA) rather than relying solely on heavy dense connections or redundant dilated convolutions. While the current model demonstrates high efficiency on workstation hardware, future work will focus on model compression and optimization to evaluate its feasibility for direct deployment on resource-constrained UAV edge devices.

### 4.3 Limitations and future directions

Architectural Ablation Scope. Although our theoretical analysis and the quantitative ablation results (**Table 1**) support the design rationale of GACA and SGF, a limitation of our current work is the absence of exhaustive empirical comparisons with closely related architectural alternatives, such as replacing the strip-pooling branch in GACA with an ASPP-style context module, or replacing SGF with simple concatenation- or addition-based fusion. This is primarily due to computational constraints. We therefore refrain from making strong claims about the absolute superiority of these specific design choices and acknowledge that targeted component-level comparisons represent an important direction for future work.

Dense Small Lesion Adhesion. Despite PlantFormer’s progress in adapting to diverse lesion morphologies, an analysis of failure cases reveals limitations, particularly when facing high-density early-stage disease outbreaks (e.g., dense rust spore pustules). In such scenarios, an Adhesion of Dense Small Lesions phenomenon was observed. This behavior highlights a broader limitation of the current “Semantic Segmentation” paradigm for fine-grained agricultural applications. If the goal is to calculate the overall Lesion Area Ratio (LAR) to guide macroscopic variable-rate spraying, the connected masks generated by PlantFormer are generally sufficient. However, if future applications require high-precision spot treatments (e.g., laser irradiation) on individual minuscule lesions, the approach may need to evolve from “Semantic Segmentation” towards “Instance Segmentation” or “Point-Supervised Counting.”

Long-tail Distribution. For extremely rare disease classes, the lack of representative features remains a bottleneck. By acknowledging these limitations, we hope to direct attention toward the next generation of fine-grained disease quantification and the future exploration of few-shot or zero-shot learning within the agricultural domain.

### 4.4 Practical implications for precision agriculture

The segmentation capability of PlantFormer holds practical value for modern field management:

- Variable-Rate Spraying (VRS): By outputting relatively noise-free disease density maps, the model shows potential to assist workstation-based systems in directing agricultural drones to apply chemicals more precisely to infected zones, aiding in the reduction of pesticide use.
- Yield Loss Prediction: The accurate calculation of the LAR provided by PlantFormer can serve as a reliable input for crop growth simulators, contributing to better estimations of potential yield loss.

## 5 Conclusion

In this paper, we introduced PlantFormer, an integrated network that adapts advanced computer vision techniques to specifically address the biological characteristics and environmental challenges of plant diseases. We identified that agricultural imagery presents specific bottlenecks for generalpurpose segmentation models: anisotropic spread of lesions, blurred biological boundaries, and pronounced background dominance.

To address these challenges, PlantFormer employs an Interactive Backbone (CLF) to preserve early details, a Global-Anisotropic Context neck (GACA) designed to capture vein-aligned disease propagation, and a Semantic-Guided Fusion decoder (SGF) acting as a feature boundary purifier. Coupled with a decoupled boundary-aware loss function, PlantFormer successfully redirects the optimization focus toward the critical necrotic transition zones.

Experiments on two distinct datasets—the unstructured PlantSeg dataset and the structured NLB dataset—demonstrate that PlantFormer achieves highly robust performance across varying field conditions, outperforming established generalist methods like DeepLabV3+ and Segformer in both quantitative metrics and visual boundary fidelity. Ultimately, PlantFormer provides a robust and interpretable visual engine for automated disease diagnosis, supporting more sustainable and precise agricultural interventions.

## Data Availability

The original contributions presented in the study are included in the article/supplementary material. Further inquiries can be directed to the corresponding authors.
